# Perturbation Analysis of a Multiple Layer Guided Love Wave Sensor in a Viscoelastic Environment

**DOI:** 10.3390/s19204533

**Published:** 2019-10-18

**Authors:** Tao Wang, Ryan Murphy, Jing Wang, Shyam S. Mohapatra, Subhra Mohapatra, Rasim Guldiken

**Affiliations:** 1 Department of Mechanical Engineering, College of Engineering, University of South Florida, Tampa, FL 33610, USA; taowang@mail.usf.edu (T.W.); ryanpatrickm@mail.usf.edu (R.M.); 2 Center for Research and Education in Nanobioengineering, University of South Florida, Tampa, FL 33612, USA; smohapat@health.usf.edu (S.S.M.); smohapa2@health.usf.edu (S.M.); 3 Department of Internal Medicine and Pharmacy Graduate Programs, University of South Florida, Tampa, FL 33612, USA; 4 Microfluidics and Acoustics Laboratory, College of Engineering, University of South Florida, Tampa, FL 33610, USA; 5 James A Haley VA Hospital, Tampa, FL 33612, USA; 6 Department of Electrical Engineering, University of South Florida, Tampa, FL 33610, USA; jingw@usf.edu; 7 Department of Molecular Medicine, University of South Florida, Tampa, FL 33612, USA

**Keywords:** surface acoustic wave, love wave, guided layers, perturbation, ZnO

## Abstract

Surface acoustic wave sensors have the advantage of fast response, low-cost, and wireless interfacing capability and they have been used in the medical analysis, material characterization, and other application fields that immerse the device under a liquid environment. The theoretical analysis of the single guided layer shear horizontal acoustic wave based on the perturbation theory has seen developments that span the past 20 years. However, multiple guided layer systems under a liquid environment have not been thoroughly analyzed by existing theoretical models. A dispersion equation previously derived from a system of three rigidly coupled elastic mass layers is extended and developed in this study with multiple guided layers to analyze how the liquid layer’s properties affect the device’s sensitivity. The combination of the multiple layers to optimize the sensitivity of an acoustic wave sensor is investigated in this study. The Maxwell model of viscoelasticity is applied to represent the liquid layer. A thorough analysis of the complex velocity due to the variations of the liquid layer’s properties and thickness is derived and discussed to optimize multilayer Surface acoustic wave (SAW) sensor design. Numerical simulation of the sensitivity with a liquid layer on top of two guided layers is investigated in this study as well. The parametric investigation was conducted by varying the thicknesses for the liquid layer and the guided layers. The effect of the liquid layer viscosity on the sensitivity of the design is also presented in this study. The two guided layer device can achieve higher sensitivity than the single guided layer counterpart in a liquid environment by optimizing the second guided layer thickness. This perturbation analysis is valuable for Love wave sensor optimization to detect the liquid biological samples and analytes.

## 1. Introduction

Biosensors are used in a broad range of applications such as clinical diagnosis [1], biomedical devices [2,3,4], food production and analysis [5,6], microbiology [7,8], pharmaceutical and drug analysis [9,10], pollution control and monitoring [11], and military applications [12,13]. Surface acoustic wave (SAW)-based biosensors have played an important role in bio-detection due to their advantage of low-cost, portability, rapid detection rate, high sensitivity, analyte selectivity, and stability [3]. SAW devices with various sensing abilities or guided layer properties have been widely explored for fluidic and under liquid environment applications [14,15,16,17,18,19]. Shear horizontal (SH) polarized wave modes have less radiation loss to the liquid environment compared to the Rayleigh surface waves due to very small wave propagation normal to the substrate [20,21]. When a guided layer of finite thickness is deposited on a semi-infinite thick substrate, the Love wave can be characterized by a slower shear wave velocity on the guided layer which can result in a very high sensitivity due to their acoustic energy concentration in the layer [22]. In that way, the Love wave sensor is mainly based on the shear horizontal wave because of its high sensitivity as compared to the traditional acoustic wave-based device designs. These waves propagate in a layered structure consisting of a substrate and a guided layer on its top, which increases the sensitivity and the coupling coefficient due to the waveguide effect [23]. Due to the high sensitivity, low cost, and capability of wireless interfacing, the surface acoustic wave-based biosensors are a promising candidate for fluidic sensor applications.

When the acoustic path is covered underneath a liquid, a part of the path is displaced by the Love waves, changing its propagation properties in terms of phase velocity of the wave and acoustic losses. The geometrical properties of the guided layer and liquid layer affect the propagation velocity of the wave and the penetration path. For highly viscous fluids, viscous damping can be a limiting factor as the acoustic energy is dissipated into the media. Previously, various mathematical and simulation methods have been developed and applied to surface acoustic wave device modeling. However, there is still room for theoretical issues to be further investigated and expanded upon [24]. A particular issue arises when optimizing and designing a highly sensitive liquid-based surface acoustic wave sensor coupled with a complex suspension liquid environment. The majority of prior case studies are focused on the three-layered device structure which limits the usefulness of the numerical model to the practical applications. In the previous studies, the viscous liquid layer was assumed to have an infinite thickness. Some of the previous studies simplify the device to be two-layer or three-layer structure [25,26,27,28]. In 1999, Marina V. Voinova introduced the three guided layer structure to the quartz crystal microbalance (substrate, guided layer, and liquid layer with infinite thickness) [29,30,31]. Then, Glen McHale developed the perturbation model on the surface acoustic device and Love wave device. In his studies, the four-layer structure (substrate, guided layer, mass layer, and liquid layer) was simplified to a three-layer structure by assuming the thickness of the mass layer is equal to zero (substrate, guided layer, liquid layer with infinite thickness) [32]. However, when a viscous solution is placed on a device with double guided layers or when there is a mass loading layer in the solution layer, the simplified three-layer sensor structure can no longer be an accurate model. Instead, the four-layer system is a prevalent sensor structure in the immunosensor design. The four-layer design with a two guided layer structure under a liquid environment has not been thoroughly analyzed by existing theoretical models. The analytical and numerical analysis of the Love wave in a liquid environment to obtain the acoustic phase velocity and attenuation or complex propagation constant has been investigated in a previous study [33]. The perturbation analysis methods are focused on the investigation of the surface acoustic wave dispersion curve, which indicates the acoustic wave propagation characteristics in a layered structure. The perturbation analysis method is a very effective way to analyze and optimize the Love wave sensor with a multilayered structure compared to other methods such as the transfer matrix method and finite element method. It has its own unique advantages such as fast analysis and less resource consumption. The perturbation theory is a valuable complementary method for the finite element method (FEM) that is much more time and resource-demanding. In our recently reported prior study, the full 3D FEM for surface acoustic wave characteristic properties and sensitivity often takes more 96 GB RAM memory and more than 72 h to obtain just one data point [34]. The comparison between different materials for the four-layer device studied herein by using the FEM and transfer matrix method will need more time and more resources. However, the perturbation analysis method needs less time, resources, and can obtain a reasonably accurate result for design optimization. Then, the perturbation result can provide valuable design insights followed by a full FEM model simulation, which could save significant time for each layer of material optimization. It is very effective to use the perturbation theory as a complementary method for the FEM, which was first shown in our previous report [14]. To quickly compare the sensitivity among different guided layers for design optimization rather than obtaining complete and best-matched responses, the perturbation analysis is one of the more affordable, insightful, and efficient solutions. In particular, the effect of the substitution of the guided layer from material A to material B on sensor responses can be determined by perturbation theory in minutes, which is challenging for other methods that are time and resource-demanding. In 1985, the perturbation theory for an acoustic wave on the elastic layer was presented [35]. McHale et. al. built a mathematical model for the Love wave on the elastic and viscoelastic layer, which also demonstrated a three-layer and a four-layer system [32]. However, the four-layer system in that study assumed that the mass layer could be ignored and then reverted to the three-layer system to solve under the restrictive boundary conditions. After that, many researchers utilized the perturbation analysis to investigate the effects of the different material properties on the dispersion curve and system sensitivity [36]. The two- and three-layer system have been investigated under a viscoelastic environment with a top liquid layer added to the system for a long time [37]. However, for an immunoassay sensor used in the liquid environment, the structure with four layers is very common. The first layer is the substrate, and the second layer is the first guided layer made of SiO_2_, ZnO, or Poly (methyl methacrylate) (PMMA). The third layer usually can be a function layer with an antibody and antigen or another metal guided layer. The fourth layer represents the liquid environment. This common configuration can be seen in many sensor devices developed for practical (often biomedical) sensing applications [38,39,40]. Until now there has not been a realistic four-layer system theoretical model that has been thoroughly investigated in a viscoelastic environment. However, the current theoretical model of two and three layer is not enough to study the four-layer structure under a viscoelastic environment [41,42,43,44]. In each of the above studies of viscoelasticity through perturbation analysis, the results have been given with limitations including ignoring the elastic mass layer and an assumption of a Newtonian liquid layer of infinite thickness. In addition, the relationship between the slope of the dispersion curve and the response of a Love wave on the second guided layer or mass layer has not been thoroughly investigated [45]. Overall, the prior studies are not detailed enough to mathematically provide comprehensive analytical results for the velocity shift and attenuation response of four-layer Love waves in the liquid environment. 

In this study, a model for a four-layer Love wave system was built to explore the relationship between the liquid properties and Love wave sensor sensitivity. An idea of viscoelasticity layer with two guided layers are applied on the device with a complex shear modulus defined. At the same time, the equations derived describe the viscoelasticity using a relaxation time and the Maxwell model to demonstrate the sensitivity and the phase velocity in the new four-layer model. This study presents a path to improve the sensitivity of the surface acoustic wave multilayer system by optimization of the thickness and material types of each guided layers, the substrate and the liquid layer.

## 2. Analytical Modeling

In this study, a traditional perturbation analysis of the surface acoustic wave on the piezoelectrical substrate is investigated with the assumption of the guided layers and liquid layer to be both an isotropic layer [35,45,46]. The surface wave is considered to be a pure mechanical wave without electron perturbation or Leaky waves. The aim of the perturbation theory is to study the contribution of the design parameters to the velocity changes after the waves generated by the substrate. Maxwell’s model of the viscoelasticity for the protein layer and glycerol solution was used in this system. The material properties used in the perturbation analysis is given in Table 1.

**Figure 1:**
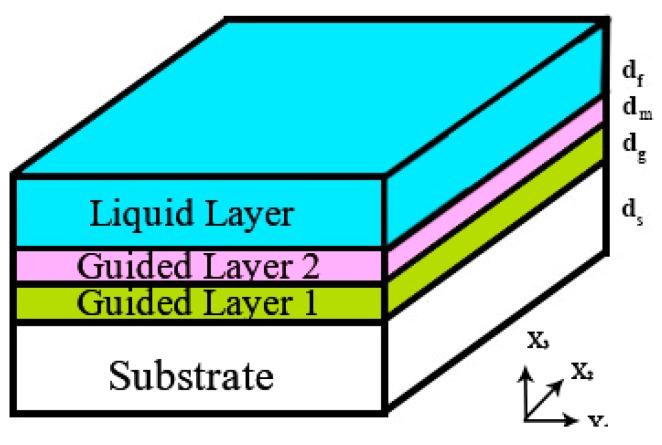
Schematic diagram illustrating the axes and layer parameters for the propagation of shear horizontally polarized acoustic waves in a four-layer system. The first layer is the substrate, and the second layer is the first waveguided layer, the third layer is the second guided layer and the fourth layer is the liquid layer.

The wave propagation was investigated for a four-layer system, as illustrated in Figure 1. The coordinate axes are placed in such a way that x_1_–x_2_ plane stays in parallel with the upper surface of the substrate and x_3_ axis is normal to this plane with x_3_ = 0 defining the substrate’s top surface.

The governing equation of motion for wave propagation in a non-piezoelectric, isotropic medium can be written as [48]:

(1) $$\frac{\rho \delta^{2}u_{j}}{\delta t^{2}}=\left(\mu +\lambda \right)\frac{\delta S_{ij}}{\delta x_{j}}+\mu \;\nabla^{2}u_{j},$$

where *u* is particle displacement, ρ is the density, *λ* and *μ* are the Lame’s constants, and *S* is the strain tensor. In previous studies, Glen McHale et al. built a model to investigate the propagation of shear horizontal acoustic waves in a system, which can consist of two layers, three layers, or four layers [31]. In one of the studies, the theoretical developments are expanded to a four-layer model; however, the liquid layer thickness and its mass layer have been ignored, therefore severely limiting the applicability of the study. In our study, the substrate, metal layer, guided layer, and mass layer were all treated as rigidly coupled elastic layers. As a result, Equation (1) of motion in each material can be further simplified as the following [32]:

(2) $$\frac{\rho \delta^{2}u_{j}}{\delta t^{2}}=\mu \;\nabla^{2}u_{j},$$

where *ρ* and μ are the density and shear modulus of the materials. Then, Equation (2) is solved in each layer using the trial solutions of the following form:

(3) $$u_{s}=\left(0,1,0\right)\left[A_{s}e^{-T_{s}x_{3}}+B_{s}e^{T_{s}x_{3}}\right]e^{j\left(\omega t-k_{1}x_{1}\right)},$$

(4) $$u_{g}=\left(0,1,0\right)\left[A_{g}e^{-jT_{g}x_{3}}+B_{g}e^{jT_{g}x_{3}}\right]e^{j\left(\omega t-k_{1}x_{1}\right)},$$

(5) $$u_{m}=\left(0,1,0\right)\left[A_{m}e^{-jT_{m}x_{3}}+B_{m}e^{jT_{m}x_{3}}\right]e^{j\left(\omega t-k_{1}x_{1}\right)},$$

(6) $$u_{f}=\left(0,1,0\right)\left[A_{f}e^{-jT_{f}x_{3}}+B_{f}e^{jT_{f}x_{3}}\right]e^{j\left(\omega t-k_{1}x_{1}\right)}.$$

The subscripts *s*, *g*, *m*, and *f* represent the substrate, guided layer, second guided layer and the fluidic layer, respectively. The *k* is a wave factor, which is defined as *ω*/*v*, where *ω* is the angular frequency and *v* is the phase speed of the wave. 

A and B are constants that determine the characteristics of the wave propagation while the *T* constants are the wave vectors. The wave propagation is only along the x_1_ direction. The trial solutions from Equation (3) to Equation (6) are substituted into Equation (2) and the following solutions of the wave vectors are then obtained:

(7) $$T_{s}^{2}=\;\omega^{2}\;\left(\frac{1}{v}-\frac{1}{v_{s}^{2}}\right),$$

(8) $$T_{g}^{2}=\;\omega^{2}\;\left(\frac{1}{v_{g}}-\frac{1}{v^{2}}\right),$$

(9) $$T_{m}^{2}=\;\omega^{2}\;\left(\frac{1}{v_{m}}-\frac{1}{v^{2}}\right),$$

(10) $$T_{f}^{2}=\;\omega^{2}\;\left(\frac{1}{v_{f}}-\frac{1}{v^{2}}\right),$$

where v_s_, v_g_, v_m_, and v_f_ are the shear velocities of the substrate, first guided layer, second guided layer, and fluid layers, respectively, while ν is the solution that characterizes the velocity of the wave in the entire system. The coordinate axes are defined such that the x_1_–x_2_ plane is parallel with the upper surface of the bottom substrate layer and x_3_ is orthogonal to the x_1_–x_2_ plane, where the upper surface of the substrate layer is positioned with x_3_ = 0. The four-layer shear horizontal wave propagation solution is found by trial solutions for propagation along the x_1_ axis and displacement in the x_2_ axis. Due to the polarization of the shear horizontal surface waves, it can be observed from the equations that the particle displacement is limited to only the x_2_-direction in the x_1_−x_2_ plane. The wave vector for the substrate layer is different than the other layers because the trial solution was chosen to ensure a zero-imaginary value of the substrate wave vector amplitude thus leading to a real substrate wave velocity value and particle displacement decaying with depth. To specify the solution, the trial solution constants from Equations (3) to (6) need to be defined and the boundary conditions due to displacement continuity between the four layers are presented as below:

(11) $$u_{s}\left(x_{3}=0\right)=u_{g}\;\left(x_{3}=0\right)\to A_{s}+B_{s}=A_{g}+B_{g},$$

(12) $$u_{g}\left(x_{3}=d_{m}\right)=u_{m}\left(x_{3}=d_{m}\right)\;\to A_{g}e^{-jT_{g}d_{g}}+\;B_{g}e^{jT_{g}d_{g}}=\;A_{m}e^{-jT_{m}d_{g}}+\;B_{m}e^{jT_{m}d_{g}},$$

(13) $$u_{m}\left(d_{g}+d_{m}\right)=u_{f}\left(d_{m}+d_{g}\right)\;\;\to \;A_{m}e^{-jT_{m}\left(d_{m}+d_{g}\right)}+B_{m}e^{jT_{m}\left(d_{m}+d_{g}\right)}=A_{f}e^{-jT_{f}\left(d_{m}+d_{g}\right)}+B_{f}e^{jT_{f}\left(d_{m}+d_{g}\right)}.$$

The stress boundary condition requires the $\tau_{i3}$ component of the stress tensor, which is shown below:

(14) $$\tau_{i3}=\;\delta_{i2}\mu \left(\frac{\delta u_{2}}{\delta x_{3}}\right).$$

The stress-free top and bottom surfaces, as well as the stress interfaces between the layers of the multilayer system, satisfy the following boundary conditions:

(15) $$\tau_{s}\left(-d_{s}\right)=0\;\to \;\;A_{s}e^{T_{s}d_{s}}-\;B_{s}e^{-T_{s}d_{s}}=0,$$

(16) $$\tau_{f}\left(d_{g}+d_{m\;}+d_{f}\right)=0\to \;A_{f}e^{-jT_{f}\left(d_{g}+d_{m}+d_{f}\right)}-\;\;B_{f}e^{jT_{f}\left(d_{g}+d_{m}+d_{f}\right)}=0,$$

(17) $$\tau_{s}\left(0\right)=\;\tau_{g}\left(0\right)\to \;\mu_{s}T_{s}A_{s}-\;\mu_{s}T_{s}B_{s}=\;j\mu_{g}T_{g}A_{g}-\;j\mu_{g}T_{g}B_{g},$$

(18) $$\tau_{g}\left(d_{g}\right)=\;\tau_{m}\left(d_{g}\right)\;\to \;ju_{g}T_{g}A_{g}e^{-jT_{g}d_{g}\;\;}-\;ju_{g}T_{g}B_{g}e^{jT_{g}d_{g}}=\;ju_{m}T_{m}A_{m}e^{-jT_{m}d_{g}}-ju_{m}T_{m}B_{m}e^{jT_{m}d_{g}},$$

(19) $$\tau_{m}\left(d_{m}+d_{g}\right)=\;\tau_{f}\left(d_{m}+d_{g}\right)\;\to A_{m}e^{-jT_{m}\left(d_{m}+d_{g}\right)}-B_{m}e^{jT_{m}\left(d_{m}+d_{g}\right)}\;=\xi_{fm}A_{f}e^{-jT_{f}\left(d_{m}+d_{g}\right)}-\xi_{fm}B_{f}e^{jT_{f}\left(d_{m}+d_{g}\right)}.$$

The above equations can be solved by using the boundary conditions with the various wave characteristics constants. The wave characteristic constants $\xi_{sg}$, $\xi_{mg}$, and $\xi_{fm\mathrm{and}\;}$are defined by *T* constants from Equations (7)–(10) and the shear modulus of $G_{s}$, $G_{g}$, $G_{f},\;$and $G_{m}$. The solved equations can be written as the following:

(20) $$\xi_{sg}=\frac{G_{s}T_{s}}{G_{g}T_{g}},$$

(21) $$\xi_{mg}=\frac{G_{m}T_{m}}{G_{g}T_{g}},$$

(22) $$\xi_{fm}=\frac{G_{f}T_{f}}{G_{m}T_{m}}.$$

The system of equations above can then be organized and re-written into matrix form as follows:

(23) $$\begin{array}{cccc} \left(\begin{array}{cccccccc} 1 & 1 & -1 & -1 & 0 & 0 & 0 & 0\\ e^{T_{s}d_{s}} & -e^{-T_{s}d_{s}} & 0 & 0 & 0 & 0 & 0 & 0\\ 1 & -1 & -j\xi_{sg} & j\xi_{sg} & 0 & 0 & 0 & 0\\ 0 & 0 & e^{-j\left(T_{g}d_{g}\right)} & -e^{j\left(T_{g}d_{g}\right)} & -\xi_{mg}e^{-j\left(T_{m}d_{g}\right)} & \xi_{mg}e^{j\left(T_{m}d_{g}\right)} & 0 & 0\\ 0 & 0 & e^{-j\left(T_{g}d_{g}\right)} & e^{j\left(T_{g}d_{g}\right)} & -e^{-j\left(T_{m}d_{g}\right)} & -e^{j\left(T_{m}d_{g}\right)} & 0 & 0\\ 0 & 0 & 0 & 0 & e^{-jT_{m}\left(d_{m}+d_{g}\right)} & -e^{jT_{m}\left(d_{m}+d_{g}\right)} & -\xi_{fm}e^{-jT_{f}\left(d_{m}+d_{g}\right)} & \xi_{fm}e^{jT_{f}\left(d_{m}+d_{g}\right)}\\ 0 & 0 & 0 & 0 & e^{-jT_{m}\left(d_{m}+d_{g}\right)} & e^{jT_{m}\left(d_{m}+d_{g}\right)} & -e^{-jT_{f}\left(d_{m}+d_{g}\right)} & -e^{jT_{f}\left(d_{m}+d_{g}\right)}\\ 0 & 0 & 0 & 0 & 0 & 0 & e^{-jT_{f}\left(d_{m}+d_{g}+d_{f}\right)} & e^{-jT_{f}\left(d_{m}+d_{g}+d_{f}\right)} \end{array}\right) & \left(\begin{array}{c} A_{s}\\ B_{s}\\ A_{g}\\ B_{g}\\ A_{m}\\ B_{m}\\ A_{f}\\ B_{f} \end{array}\right) & = & \left(\begin{array}{c} 0\\ 0\\ 0\\ 0\\ 0\\ 0\\ 0\\ 0 \end{array}\right) \end{array}$$

To avoid solving for a non-trivial solution, the determinant of the matrix is to be set to zero and the resulting velocities that satisfy the above condition are the phase velocities of the multilayer system. It is also important to note that the liquid and mass layer as defined in the context of the SAW sensor application is anticipated to exhibit viscoelastic behaviors, which can be described as the transition from an elastic solid to a viscous fluid when working under liquid loading. The complex shear modulus $G_{f}$ in Equation (24) is introduced to the system via Maxwell’s model of viscoelasticity [49,50,51]:

(24) $$G_{f}=\frac{j\omega \eta_{f}}{1+j\omega \tau }$$

where *ω* = *2πf* and $\tau =\eta_{f}/\mu \;$so that η is the fluid viscosity and τ represents the relaxation time or the time duration that it takes for the perturbed layer to return to equilibrium. It is important to note that *ωτ* approaches infinity for an elastic solid and 0 for a Newtonian fluid [52]. In this study, the *ωτ* is considered to be 10^6^ and 10 for the elastic solid layer and the Newtonian fluid layer, respectively.

Through simplification and rearrangement of terms, the four-layer dispersion matrix above can be simplified to [32]:

(25) $$\begin{array}{ll} \left[\tan\left(T_{g}d_{g}\right)-\xi_{sg}\tanh\left(T_{s}d_{s}\right)\right] & +\xi_{fg}\tan\left(T_{f}d_{f}\right)\left[1+\xi_{sg}\tan\left(T_{g}d_{g}\right)\tanh\left(T_{s}d_{s}\right)\right]\\ & =\tan\left(T_{m}d_{m}\right)\{\tan\left(T_{f}d_{f}\right)\xi_{fm}\left[\tan\left(T_{g}d_{g}\right)-\xi_{sg}\tanh\left(T_{s}d_{s}\right)\right]\\ & -\xi_{mg}\left[1+\xi_{sg}\tan\left(T_{g}d_{g}\right)\tanh\left(T_{s}d_{s}\right)\right]\} \end{array}$$

where any *ξ_ij_* value is defined in the Equation (20) to Equation (22) due to the viscoelastic properties of the materials.

When the third layer is set to zero by assuming very small perturbations ($d_{m}=0)$ in Equation (25) as reported in McHale’s study, the right-hand side becomes zero [32]. However, when ignoring the mass layer, the second layer’s properties and liquid layer’s thickness will have no contribution to the dispersion curve, which is to be addressed further herein. In this study, a thorough perturbation analysis of a four-layer is presented, which takes the mass loading layer into account. Considering the usage of new terms$\;x=T_{g}d_{g}$, $\beta =\omega d_{g}\sqrt{\frac{1}{v_{g}^{2}}-\frac{1}{v_{s}^{2}}}$, $\gamma =\omega d_{f}\sqrt{\frac{1}{v_{f}^{2}}-\frac{1}{v_{g}^{2}}}$, $\alpha =\omega d_{m}\sqrt{\frac{1}{v_{m}^{2}}-\frac{1}{v_{g}^{2}}}$, and using the identity, Equation (25) can be simplified to:

(26) $$\begin{array}{ll} \tan x-\left(\frac{\mu_{s}}{G_{g}}\right)\sqrt{\left[{\left(\frac{\beta }{x}\right)}^{2}-1\right]} & \tanh\left(\frac{d_{s}}{d_{g}}\sqrt{\beta^{2}-x^{2}}\right)+\left(\frac{G_{g}}{\mu_{g}}\right)\sqrt{\left[{\left(\frac{\gamma }{x}\right)}^{2}+1\right]}\tan(\frac{d_{f}}{d_{g}}\sqrt{\gamma^{2}+x^{2}})*\left[1+\left(\frac{\mu_{s}}{G_{g}}\right)\sqrt{\left[{\left(\frac{\beta }{x}\right)}^{2}-1\right]}\tan x\tanh\left(\frac{d_{s}}{d_{g}}\sqrt{\beta^{2}-x^{2}}\right)\right]\\ & -\tan(\frac{d_{m}}{d_{g}}\sqrt{\alpha^{2}-x^{2}})[\tan\left(\frac{d_{f}}{d_{g}}\sqrt{\gamma^{2}+x^{2}}\right)*\left(\frac{G_{f}}{\mu_{m}}\right)*\frac{\sqrt{\left[{\left(\frac{\gamma }{x}\right)}^{2}+1\right]}}{\sqrt{\left[{\left(\frac{\alpha }{x}\right)}^{2}-1\right]}}*\left(\tan\left(x\right)-\left(\frac{\mu_{s}}{G_{g}}\right)\sqrt{\left[{\left(\frac{\beta }{x}\right)}^{2}-1\right]}*\tanh\left(\frac{d_{s}}{d_{g}}\sqrt{\beta^{2}-x^{2}}\right)\right)\\ & -\left(\frac{\mu_{m}}{G_{g}}\right)\sqrt{\left[{\left(\frac{\alpha }{x}\right)}^{2}-1\right]}*(1+\left(\frac{\mu_{s}}{G_{g}}\right)*\sqrt{\left[{\left(\frac{\beta }{x}\right)}^{2}-1\right]}\tan x\tanh\left(\frac{d_{s}}{d_{g}}\sqrt{\beta^{2}-x^{2}}\right))]=0. \end{array}$$

Numerical approximations using the Matlab can be used to iteratively solve for the guided layer wave dispersion phase velocities. This real phase velocity represents the first Love wave mode that has been discussed in detail elsewhere [14,16,53].

The sensitivity of the liquid layer on top of the two guided layers and substrate can be analyzed by Equation (27) [54]. The derivation and expansion of the Equation (27) is shown in the supplementary Equations (28) and (29):

(27) $$S_{m}^{fluid}=\;\underset{dm}{\lim}\frac{1}{\rho_{f}d_{f}}{\left(\frac{dv}{dx}\right)}_{x4=d_{f}}Re{\left(\frac{\Delta v}{v}\right)}^{fluid}$$

(28) Sr=limΔx→0Δffm=limΔx→0Δvvm=limdm1ρfdf(dvdx)x4=dfRe(Δvv)fluid

(29) $$\frac{\Delta v}{v_{0}}=\left[\frac{1+\xi_{fm}{}^{2}\tan^{2}\left(T_{f}d\right)}{1+\xi_{fg}{}^{2}\tan^{2}\left(T_{f}d\right)}\right]\left(\frac{1-v_{m}^{2}/v_{0}^{2}}{1-v_{g}^{2}/v_{0}^{2}}\right)\times \left(\frac{d\log_{e}v}{dz}\right)\frac{\omega \rho_{m\Delta h}}{2\pi v_{g}\rho_{g}}$$

## 3. Results and Discussion

This study aims to investigate how the thicknesses and properties of each layer influence the sensitivity in a four-layer design with multiple guided layers and a liquid layer on top. The remainder of the results and discussion is organized as follows. 

First, a study of the three-layer system without a liquid layer is investigated to verify and compare with prior studies before initiating a study of the four-layer system. Then, the study focuses on the thickness of the guided layer’s effect on the sensitivity. Thereafter, the effect of the liquid layers’ thickness on the phase velocity and sensitivity is also investigated and presented. At last, a detailed investigation on how the viscosity of the liquid layer affects the sensitivity is demonstrated.

### 3.1. Second Guided Layer Material Effect on Phase Velocity and Sensitivity

**Figure 2:**
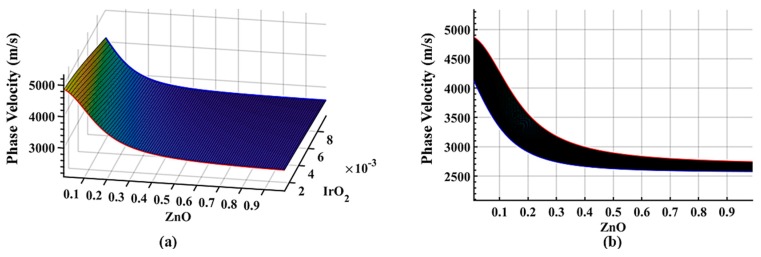
Dispersion surface plots of the three-layer system with a quartz substrate as the first layer, ZnO guided layer as the second layer, IrO_2_ guided layer as the third layer, and no liquid layer at 100 MHz with relaxation time, ωτ = 10^6^, showing (**a**) a phase velocity plot in isometric view; and (**b**) a plot of phase velocity vs the variation of ZnO layer thickness.

A three-layer vacuum system is obtained from Equation (25) without a fluid layer by setting the value of *d_f_* to 0. Figure 2a,b illustrate the phase velocity dispersion. However, to compare all the parametric studies, we simplified the mathematical problem by only solving the first mode of the propagated wave with an operating frequency of 100 MHz on a substrate with a thickness of 500 µm. Figure 2 shows the solution of the phase velocity as a function of the normalized two guided layers thicknesses (ZnO and IrO_2_ layer). Z_g_, Z_m_ represent are the non-dimensional normalized thickness of the guided layers in the x-axis and y-axis. The non-dimensional thickness is defined as $Z_{i}=d_{i}*f/v_{i}^{\infty }$, where $d_{i}$ is the thickness, *f* the operating frequency, and $v_{i}^{\infty }$ is the shear velocity in each guided layer. As shown in Figure 2, the initial phase velocity of the system is highly dependent on the thickness of the ZnO and IrO_2_ layer. A prior study has discussed this sensitivity transition [36,55]. With less phase velocity difference between the substrate and guided layer, the subtle transition results in a lower sensitivity. The dispersion curve of the red and blue represent design with an IrO_2_ layer with a non-dimensional thickness of 0.001 and 0.01. As shown in Figure 2b, as the thickness of the ZnO layer increases, the velocity approaches the ZnO shear velocity, which shows how the Love waves perturbate at the ZnO layer. Also, if the ZnO layer thickness is held constant while increasing the IrO_2_ layer thickness, the phase velocity decreases and approaches the shear velocity of the IrO_2_ guided layer. Thus, the thicker the IrO_2_ layer, the lower the initial phase velocity will be.

**Figure 3:**
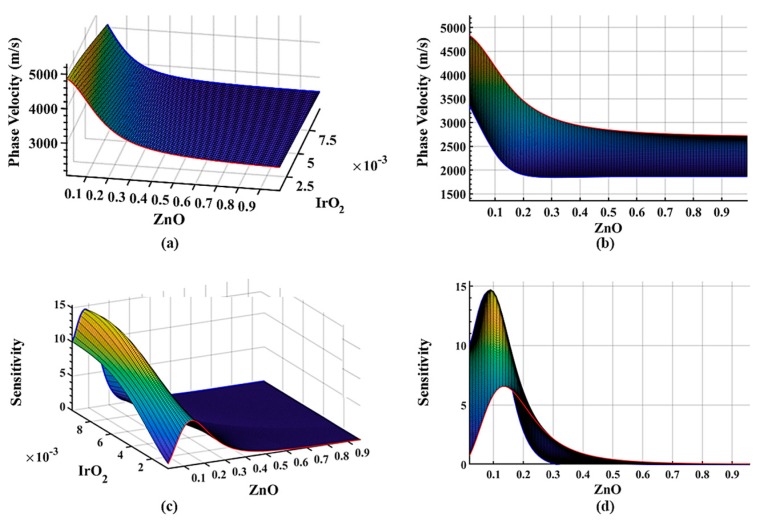
Dispersion surface plots of the four-layer system with a quartz substrate as the first layer, ZnO guided layer as a second layer, IrO_2_ guided layer as the third layer, and a liquid layer normalized thickness of 0.025 at 100 MHz with relaxation time, *ωτ* = 10. The normalized thickness of the fluid layer is 0.025 with a constant viscosity of 20 cP. (**a**) Isometric view of the phase velocity dispersion with varying thicknesses of the first and second guided layers; (**b**) phase velocity vs. ZnO thickness variation; (**c**) isometric view of the mass sensitivity (unit: m^2^/kg) dispersion with varying thickness of the first and second guided layers; (**d**) sensitivity (unit: m^2^/kg) vs. the variation of the ZnO layer thickness.

Figure 3 illustrates the dispersion surface plots of the four-layer system with a quartz substrate as the first layer, ZnO guided layer as a second layer, IrO_2_ guided layer as the third layer, and a liquid layer of a normalized thickness of 0.025 at 100 MHz with relaxation time, ωτ = 10. As shown in Figure 2 and Figure 3, it is obvious that the dispersion curve of the phase velocity decreases by the introduction of the viscoelastic c layer after the liquid layer is applied to the surface. This shows that the phase velocity is approaching the shear velocity of the liquid layer. The sensitivity of the system also decreases due to the viscous damping of the liquid layer. The phase velocity of the system with a thicker IrO_2_ layer is more sensitive to the liquid due to decreased phase velocity by the viscoelastic layer as seen in Figure 3c,d. A design with a thicker IrO_2_ layer is more affected by the liquid layer as can be seen in Figure 3a,b. And as the thickness of the IrO_2_ layer increases, the dispersion curve has a sharp transition to a lower velocity, which can be observed by comparing the red curve and blue curve in Figure 3a,b. One can observe from Figure 3 that the maximum sensitivity is 14.7 m^2^/kg. The maximum mass sensitivity of the ST-cut quartz Love sensor is 45 m^2^/kg without the liquid layer [56]. With the sensor immersed into the liquid environment, the mass sensitivity can be reduced to 4 m^2^/kg ~ 30 m^2^/kg [57,58]. This model is focusing on the viscosity changes with ignoring the mass changes in the liquid layer which resulted in a lower sensitivity than some of the previous models.

The second guided layer, IrO_2_, is deposited on top of the ZnO guided layer to serve as an intermedia guided layer. However, most of the prior studies and analysis methods treat the second guided layer as a liquid mass layer, which may not fully represent the function of the second guided layer. This study brings the theoretical study on multiple guided layers in perturbation analysis to improve the sensitivity and optimize the design parameters.

### 3.2. Fluid Layer Material Effect on Phase Velocity and Sensitivity

**Figure 4:**
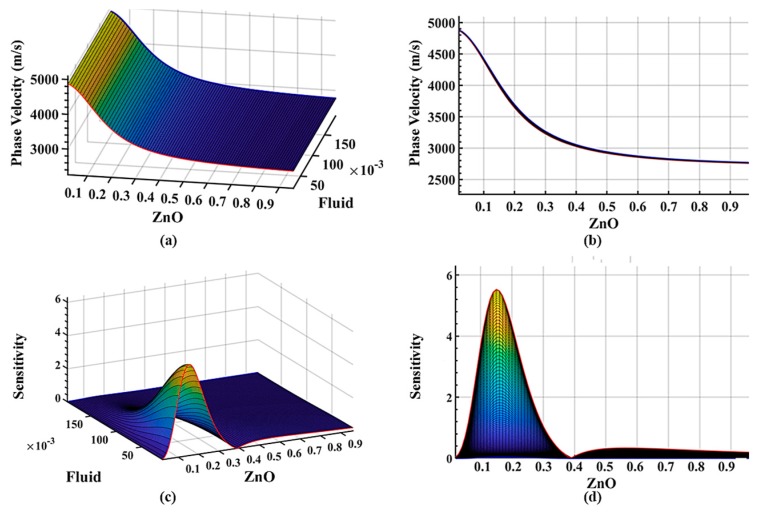
Dispersion surface plots of the four-layer system with a varying thickness of the liquid layer at 100 MHz with relaxation time, *ωτ* = 10. The thickness of the second guided layer IrO_2_ is set to 35 nm with a constant viscosity of 20 cP. (**a**) Isometric view of the phase velocity dispersion with varying thickness of the liquid layer; (**b**) phase velocity vs. ZnO thickness variation; (**c**) isometric view of the mass sensitivity (unit: m^2^/kg) dispersion with varying thickness of the liquid layer; (**d**) sensitivity (unit: m^2^/kg) vs. ZnO layer thickness variation.

How the liquid layer affects the sensitivity has not been fully investigated until recently. The effect of the liquid layer thickness on the phase velocity and sensitivity is investigated to optimize the device performance when operating under a liquid environment. An IrO_2_ layer thickness of 35 nm is retained in the parametric study, while the thickness of the liquid layer is varied. The non-dimensional thickness of the liquid layer thickness is varied from 0 to 0.2 and is applied to the four-layer system with a default liquid viscosity of 20 cP and a density of 1350 kg/m^3^. With a fixed thickness of the IrO_2_ guided layer, the thickness of the liquid only slightly affects the perturbation velocity, which is the result of the Love waves focusing energy on the guided layer. This has also been observed in Figure 4a,b. However, the thickness of the liquid layer does affect the sensitivity of the whole system as evidenced by a decrease in the system sensitivity with an increase in the non-dimensional thickness of the liquid layer as shown in Figure 4c,d. With a liquid thickness over 3 µm, the sensitivity is close to zero, which provides an insightful guideline to design a suspension media layer for sensing and detection. 

### 3.3. Viscosity Sensitivity of the Four Layer Structure

**Figure 5:**
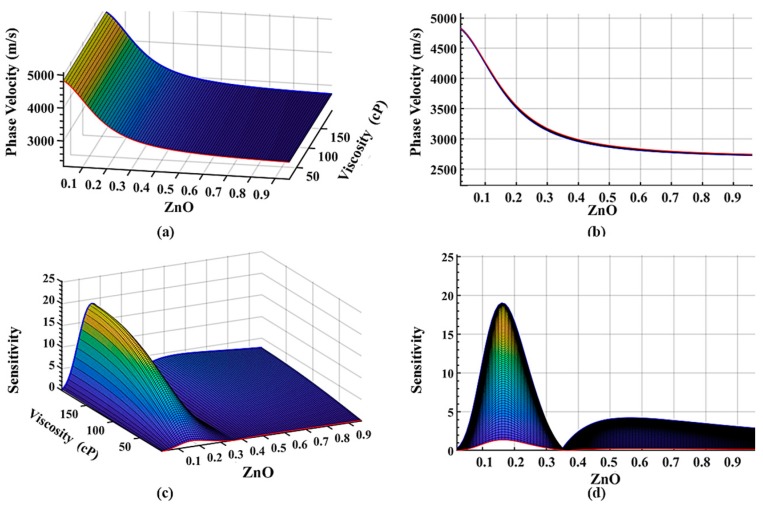
Dispersion surface plots of the four-layer system with varying viscosity of liquid layer at 100 MHz with relaxation time, *ωτ* = 10. The thickness of the second guided layer IrO_2_ is set to 35 nm, and the thickness of the liquid is chosen to be 0.1 µm. (**a**) Isometric view of the phase velocity dispersion with varying viscosity of the liquid layer; (**b**) phase velocity vs. views normal to ZnO layer thickness variation; (**c**) isometric view of the mass sensitivity (unit: m^2^/kg) dispersion with variation viscosity of the liquid layer; (**d**) sensitivity (unit: m^2^/kg) vs. view normal to ZnO layer thickness variation.

Properties of the liquid such as the viscosity and density can affect the sensitivity of the system. The sensitivity as a function of the viscosity is shown in Figure 5. The plotted sensitivity curve clearly shows that the increment of the viscosity of the liquid layer increases the sensitivity until the sensitivity reaches the saturation point. The sensitivity increase rate versus viscosity (per cP) decreases when the viscosity is higher than about 60 cP. As compared to the prior published studies [35], a similar result is observed in our study. This saturation point can be found in many other studies [59,60]. By choosing higher operation frequencies, this saturation effect can be moved to lower viscosities [60].

### 3.4. Verification of The Mass Sensitivity on Multilayer Structure

#### 3.4.1. Surface Acoustic Device Design

**Figure 6:**
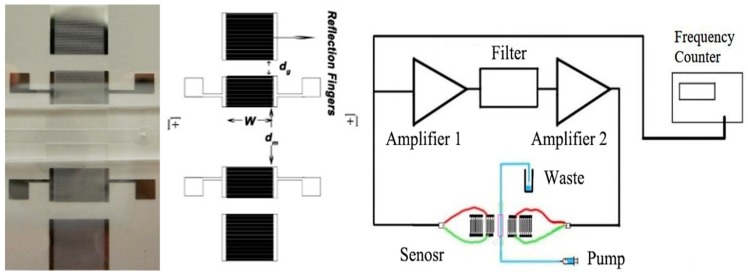
Fabricated two-port resonator on 90° Y-propagated ST-cut quartz wafers. Conceptual view of the oscillatory circuit system for detection.

A two-port surface acoustic device with a built-in PDMS microfluidic channel was fabricated and tested to compare the measured frequency response with a calculated response based on the perturbation analysis as shown in Figure 6. The electrodes of input and output inter-digited transducers (IDTs) were patterned using the standard photo-lithography reported in the previous study [15]. The devices are fabricated with a 100 nm ZnO layer on top of a 90° rotate Y propagated ST-cut quartz substrate with a thickness of 500 µm (other device parameters are illustrated in Table 2). An oscillator circuit setup was used to measure the frequency response under the different concentrations of the glycerol solution, which was reported in our previous study [14,15,21]. 

#### 3.4.2. Materials

90° Y-propagated ST-cut quartz wafers were purchased from University Wafer Inc (South Boston, MA, USA) and two gain RF amplifiers (Olympus 5073PR and Olympus 5072PR) were purchased from Olympus NDT Inc (Santa Clara, CA, USA). A digital frequency counter Agilent 53220A was purchased from Agilent Technologies Inc (Santa Clara, CA, USA) and an oscillator Tektronix TS2001C was purchased from Tektronix Inc (Beaverton, OR, USA). BenchVue Universal Counter software was licensed by Keysight Technologies (Santa Clara, CA, USA). Glycerol analytical standard solution was purchased from Sigma-Aldrich (Milwaukee, WI, USA), Slygard@184 Silicone Elastomer kit was purchased from Dow Corning Inc (Auburn, MI, USA). Mixed glycerol solution (glycerol with Di-water) was injected into the channel from the inlet by an external syringe pump (KDS200, KD Scientific, Holliston, MA, USA)

#### 3.4.3. Experiment Protocol of Measurement

After the mixed glycerol solution was pumped to the microfluidic channel, the frequency counter measures the frequency shift and transfers the real-time data to the computer. After the experiment was done, the mixed glycerol solution was removed, and the microfluidic channel was washed with three changes of deionized-water to clean the sensing area.

#### 3.4.4. Comparison of Results

**Figure 7:**
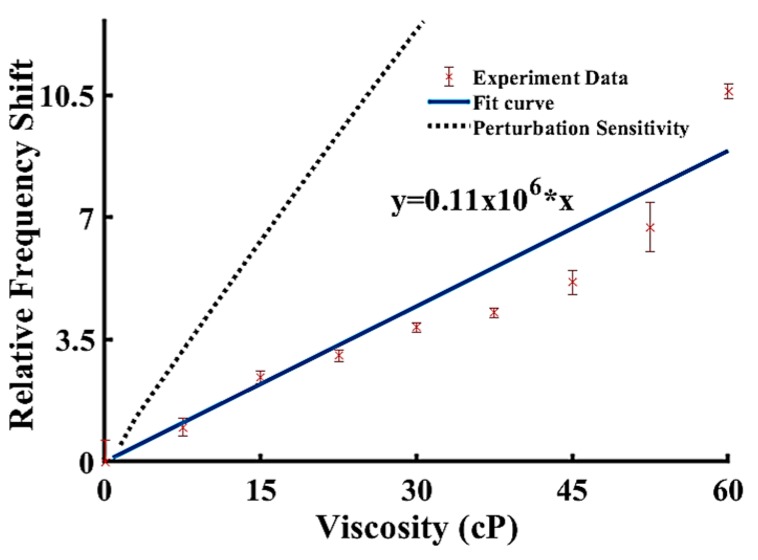
Sensitivity (frequency shift) comparison between the experiment results and model predictions of varying viscosity glycerol solution.

Figure 7 presents a comparison between the experimental results and the theoretical model predictions on the dependence of the frequency shift versus the viscosity of glycerol solution for SAW devices fabricated on a ZnO/ST-cut quartz substrate. As shown in Figure 7, the sensitivity curve predicted by the current theoretical model matches the trend of the experimental data. However, due to the limitation of the mathematical model, only some of the properties and design parameters can be interpreted by the analytical model. The model predicted that the perturbation sensitivity obtained in this study is larger than the sensitivity extracted by the experimental data. This mathematical model is a 1D study to predict the sensitivity between the design which does not include some design parameters such as the damping loss, coupling loss, and dielectric loss from the system. The mass loading effect of the microfluidic channel and the damping loss of the viscous liquid has not been taken into account by the simplified model, which may result in the discrepancy between the experiment results and perturbation analysis results. The coupling loss after the wave leaked into the liquid and generated the longitudinal wave is also not taken into account which may affect propagated wave and reduce the actual frequency shift in the experiment. The wave reflection from the propagation path, such as PDMS sidewall reflection and substrate side edges reflection, is also not taken into account to the sensitivity. However, this wave reflection at the surface will have interference with the propagated wave to reduce the realistic sensitivity. The aim of this study is to build a mathematical model to select different materials for a multiple guided surface acoustic wave device. This model has a good accuracy of the frequency shifts between experiment and analytical analysis. The perturbation theory is a faster method to compare wide variety of materials and provide approximate thickness for each layer to include the liquid layer for design optimization. As a valuable complementary method for the finite element method, the perturbation theory can narrow down the materials selection and thickness design for finite element methods as shown in our previous work [14]. 

## 4. Conclusions

It has been widely demonstrated that the Love wave sensor can sense the properties of a liquid layer. However, the relationship between the liquid properties and Love wave sensor sensitivity has not been thoroughly and analytically studied. This study presents a plausible path to increase the surface acoustic multilayer system sensitivity by a judicious design, which includes the optimization of the thickness and material types of each guided layers, substrate, and the liquid layer. Maximum sensitivity can be achieved by adjusting the thicknesses and other properties of the first and the second guided layers. Moreover, the effects of the thickness and properties of the liquid layer on top of the guided layers are investigated, which strongly affects the sensitivity of the four-layer design. The sensitivity can be increased and maximized by optimizing thickness and the viscosity of the liquid layer. Our studies have successfully demonstrated a numerical method to optimize the liquid loaded Love wave device, which indicates that the sensitivity will be close to zero when a liquid layer with a normalized thickness of over 0.1 is applied, whereas a normalized ZnO layer thickness of 0.15 is viewed as the optimal thickness. This study brings a four-layer device configuration composed of double guided layers under a liquid environment which can be applied to the future immunosensor sensor designs.

## Figures and Tables

**Table 1:** Properties for various materials that are used in the model.

Material	*E* (GPa)	*ν*	*G* (GPa)	$\rho \;(\frac{\mathrm{kg}}{m^{3}})$	*Vs* (m/s)
ST-cut 90°X Quartz				2650	4996
ZnO	90	0.15	39	5610	2650
SiO_2_	70	0.17	30	2650	3726
IrO_2_	290	0.3381	108	11660	3043
Liquid Layer			0.52 [47]	1350	35

**Table 2:** Device parameters used for the fabrication of the Interdigital transducer (IDT) transducers.

Parameters		Parameters	
Wavelength (λ)	300 μm	Channel height	100 μm
Pairs of fingers	30	Channel Length	25 mm
Pairs of reflecting fingers	50	Finger height	100 nm
Finger width	75 μm	Phase velocity	4996 m/s
Aperture (w)	9.8 mm	Wavelength of reflecting fingers	300 μm
Channel width	2 mm	Design frequency	~16.6 MHz
